# CD4^+^CD45RA^−^FOXP3^low^ Regulatory T Cells as Potential Biomarkers of Disease Activity in Systemic Lupus Erythematosus Brazilian Patients

**DOI:** 10.1155/2018/3419565

**Published:** 2018-06-12

**Authors:** Helena L. Silva-Neta, Maria C. A. Brelaz-de-Castro, Mardonny B. O. Chagas, Henrique A. Mariz, Rodrigo G. de Arruda, Viviane F. de Vasconcelos, Michelly C. Pereira, Audrey Romano, Ivan R. Pitta, Claudia D. L. Marques, Angela L. B. P. Duarte, Moacyr J. B. M. Rêgo, Maira G. R. Pitta

**Affiliations:** ^1^Laboratório de Imunomodulação e Novas Abordagens Terapêuticas (LINAT), Núcleo de Pesquisa em Inovação Terapêutica Suely Galdino (NUPIT-SG), Universidade Federal de Pernambuco (UFPE), 50670-901, Brazil; ^2^Laboratório de Parasitologia, Centro Acadêmico de Vitória, Universidade Federal de Pernambuco (UFPE), 50670-901, Brazil; ^3^Hospital das Clínicas, Universidade Federal de Pernambuco (UFPE), 50670-901, Brazil; ^4^Faculdade Nova Roma, 50830-260, Brazil; ^5^Centre for Immunology & Infection, Department of Biology and Hull York Medical School, University of York, YO10 5DD, UK

## Abstract

Heren, we analyzed Treg cells as potential biomarkers of disease activity in systemic lupus erythematosus (SLE) patients. Peripheral blood mononuclear cells from 30 SLE patients (15 active: SLEDAI > 6/15 SLE remission: SLEDAI< 6) and 15 healthy volunteers were purified. Treg immunophenotyping was performed using CD4, CD25, CD45, CD127, and FOXP3 markers. CD4^+^FOXP3^+^ Treg activation state was investigated based on CD45RA and FOXP3 expression. To increase the accuracy of our findings, a multivariate linear regression was performed. We showed a significant increase in the frequency of CD4^+^FOXP3^+^ Treg cells in SLE patients. However, unlike all other Treg cells phenotypes analyzed, only eTreg (CD4^+^FOXP3^high^CD45RA^−^) (p=0.01) subtype was inversely correlated with disease activity while Foxp3^+^nontreg (CD4^+^FOXP3^low^CD45RA^−^) (p=0.003) exerted a direct influence in the outcome of the disease. Foxp3^+^nontreg cells were the most consistent SLE active indicator, confirmed by multiple linear regression analyses. In summary, our results demonstrate Foxp3^+^nontreg cells as new biomarkers in the search of an effective therapeutic strategy in SLE.

## 1. Introduction

Systemic Lupus Erythematosus (SLE) is an autoimmune disease characterized by the presence of antibodies against self-antigens. SLE evolves by unpredictable episodes of intense inflammatory activity and remission, with localized or systemic damage [1–3].

In clinical practice, treatment depends on the manifestations of the disease; usually corticosteroid and immunosuppressant drugs are used. However, in a long-term treatment, patients become refractory to these conventional drugs. This can reduce chances of controlling the disease activity and increases death risk [4].

Searching for new therapeutic strategies for autoimmune diseases, regulatory T cells (Tregs) have a prominent place [5]. Tregs cells play a key role in maintaining self-tolerance and suppression of deleterious immune responses to patients. Abnormalities in peripheral tolerance mechanisms mediated by these cells are found in various autoimmune diseases [5–8].

SLE pathogenesis is related to defects in Treg cell homeostatic control [9–11]. Therefore, the disease development may be the result of an imbalance in the immune system effector and regulatory T cells. This imbalance is associated with an inadequate number, phenotype, or defective function of Treg cells observed in SLE [8, 12].

FOXP3 is a key transcription factor for Treg pathway. Cells termed “naturally occurring Treg cells” (nTreg or tTreg) that can be identified by the phenotype CD4^+^FOXP3^+^CD25^+^CD127^−^ are widely studied in SLE; however, conflicting results are reported in their definition and function [13, 14]. Some studies show poor suppressive ability; others do not confirm these data or reflect a wide variability among the subtypes studied [15–22]. In addition, different activation status of CD4^+^FOXP3^+^ Treg cell can be investigated based on the expression of CD45RA and FOXP3. Thus, cells having CD4^+^FOXP3^low^CD45RA^+^ phenotype set the resting cells group (naïveTreg), CD4^+^FOXP3^high^CD45RA^−^ the effector cells (eTreg), and CD4^+^FOXP3^low^CD45RA^−^ the Foxp3^+^nontreg cells (Foxp3^+^nontreg) that have showed no suppressive potential, different from the others CD4/FOXP3/CD45RA subtypes [12].

Based on the SLE complexity, all percentage of Treg cell subpopulations and their functions vary according to the severity of the disease and the therapeutic course taken [23, 24]. However, the influences of Treg cell subtypes on SLE activity remain poorly understood. Further investigation on Treg cells subsets involved in the different clinical outcomes will contribute to define new therapeutic strategies in SLE [5, 23, 24]. Therefore, in this study, we investigated the different Treg cells subsets in SLE patients with variable clinical features.

## 2. Materials and Methods

### 2.1. Patients and Control

Thirty patients (28 women and 2 men) with an average age of 35.33 (±10.40) years were invited to participate in this study. The control group consisted of 15 healthy women with an average age of 34.19 (±11.16) without diagnosis for autoimmune disease. All patients were recruited from the Rheumatology Service of Hospital das Clinicas, at the Federal University of Pernambuco, and met the classification criteria for SLE of the American College of Rheumatology [25]. Exclusion criteria were as follows: patients who refused to sign the free and informed consent term, pregnancy, comorbidities, and patients who had done pulse therapy with methylprednisolone (in the last month prior to sample collection) or used high dose steroids (greater than or equal to 1mg/kg/day of prednisone). Clinical, laboratory, and demographic parameters were assessed and are summarized in Table 1. The activity index of the disease: SLE disease activity index 2000 (SLEDAI- 2K) was used to measure the activity of SLE. Patients with SLEDAI≥ 6 were considered active (n=15), with SLEDAI≤ 6 being in remission (n=15) [26]. All patients and healthy volunteers who participated in this study signed a consent form approved by the Ethics Committee of the Federal University of Pernambuco, Brazil (CAAE–01420172000-09).

### 2.2. Laboratory Parameters

Serum samples, obtained from patients' peripheral blood, were stored at −80°C until use. Anti-dsDNA analysis was performed by indirect immunofluorescence with* Crithidia luciliae* substrate using Inova Diagnostics kit (San Diego, USA). C3 and C4 complement factors were evaluated by Immunoturbidimetry Technique (Roche Diagnostics GmbH, Mannheim, Germany).

### 2.3. Purification of Mononuclear Cells from Peripheral Blood (PBMC)

The peripheral blood collected in heparin tubes was directly added to Ficoll-Hypaque gradient (Amersham Biosciences, Uppsala, Sweden) in 50 mL falcon tube. After centrifugation at 400 x g for 40 min at 22°C, peripheral blood mononuclear cells (PBMCs) were recovered and washed twice with PBS (Phosphate-buffered saline) (pH 7.2) at 350 x g for 15 min. The PBMCs were then resuspended in RPMI 1640 medium (Roswell Park Memorial Institute) (Gibco, Thermo Fisher Scientific) supplemented with L-Glutamine, 10% Fetal Bovine Serum (Lonza), 10 mM HEPES (4- (2-hydroxyethyl)-1-piperazineethanesulfonic acid) (Gibco, Thermo Fisher Scientific), and 200 U/ml Penicillin/Streptomycin (Gibco, Thermo Fisher Scientific). An aliquot of these cells was removed for counting on a Neubauer chamber using Trypan blue (Sigma, St. Louis, MO) as a viability dye.

### 2.4. Determination of Cell Phenotypes of Treg Cells

10^5^ PBMCs were resuspended in 100uL of PBS for labeling with human cell-surface antibodies (all from eBioscience) in two different conditions: (1) antiCD25 PECy7 (BC96), antiCD127 PerCPCy5.5 (eBioRDR5), antiCD4 FITC (RPA-T4;) together, or (2) antiCD4 FITC (RPA-T4) and antiCD45RA PerCPCy5.5 (HI100). Cells were then permeabilized with the “Human FoxP3 Buffer Set” BD-Pharmingen (San Diego, CA) according to the manufacturers' recommendation and labeled with FOXP3 PE (236A). A hundred thousand events per sample were acquired by Attune® (Thermo Fisher Scientific) flow cytometer. Analysis was done with FlowJo 7.6.5 (Tree Star® Inc.) (Figure 2). Previous antibody titrations and FMO (Fluorescence Minus One) control were also performed, ideal for showing gating boundaries in multicolor flow cytometry [27](Figure S1).

### 2.5. Statistical Analysis

Data analysis that did not follow the normal distribution was performed using univariate comparisons through nonparametric tests (Mann–Whitney, Kruskal-Wallis, and Kolmogorov-Smirnov). For data that followed the normality, we apply parametric tests (*t*-test or one-way ANOVA). Our data was plotted with the GraphPad Prism® version 6.0 (La Jolla, USA) and the results were set considering the median value, maximum and minimum. Multiple linear regression analyses were used to increase the accuracy of dependent correlations of two or more variables. The F-test was properly applied to validate the correlations with multiple variables, being considered p <0.05 significant for all tests.

## 3. Results

### 3.1. Naturally Occurring Treg Cells in SLE Patients and Healthy Donors

**Figure 1:**
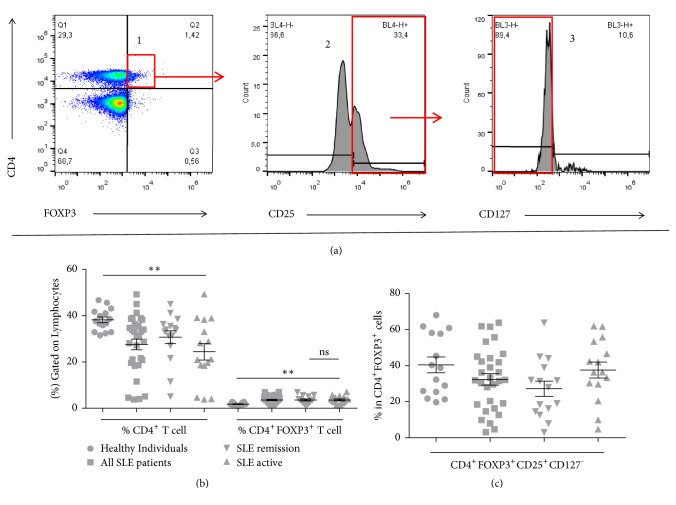
Naturally occurring Treg lymphocytes of SLE patients and healthy individuals. (a) CD4^+^FOXP3^+^CD25^+^CD127^−^ Phenotyping: CD4^+^FOXP3^+^ cells (1), CD4^+^FOXP3^+^CD25^+^ cells (2), CD4^+^FOXP3^+^CD25^+^CD127^−^ in CD4^+^FOXP3^+^ cells (3); (b) CD4^+^ T Lymphocytes and CD4^+^FOXP3^+^ Treg cells; (c) CD4^+^FOXP3^+^CD25^+^CD127^−^ cells in CD4^+^FOXP3^+^ Treg cells. ^*∗∗*^p≤0.002.

We investigated naturally occurring Treg cells in SLE patients and healthy donors according to Figure 1(a). Patients with active disease or remission had the lower proportions of CD4^+^ T lymphocytes (21.10%, 3.76-49.20% and 33.9%, 5.12-45%, respectively) compared to the healthy individuals (37.6%; 31.5-46.7%) (Figure 1(b)). A significant reduction was recorded in patients in the active disease group (p<0.001) compared to controls (Figure 1(b)). There was a significant increase in CD4^+^FOXP3^+^ Treg cells of the patients with active disease (3.73%, 1.31-7.01%) and remission (3.54%, 1.39-6.97%) in contrast to the healthy individuals (1.63%, 1.05-2.79%) (p=0.003). However, this phenotype did not vary according to the disease activity (p> 0.05) (Figure 1(b)). For a more specific T regulatory cell profile analysis, CD25 and CD127 expressions were also evaluated in the CD4^+^FOXP3^+^ Treg cells. For the CD4^+^FOXP3^+^CD25^+^CD127^−^ Treg cell profile, there were no significant variations among groups of patients with active SLE (36.38%, 4.70-61.80%), remission (28.12%, 3.12-63.83%) or healthy subjects (38.80%, 19.72-68.07%) (p>0.05) (Figure 1(c)). Likewise, there was no significant difference between all SLE patients (32%; 31.23-63.83%) and healthy subjects group (p>0.05) (Figure 1(c)).

### 3.2. CD4^+^CD45RA^−^FOXP3^low^ (Foxp3^+^nonTreg) and CD4^+^CD45RA^+^FOXP3^low^ (naïve Treg) T Cells Increased in SLE Patients

**Figure 2:**
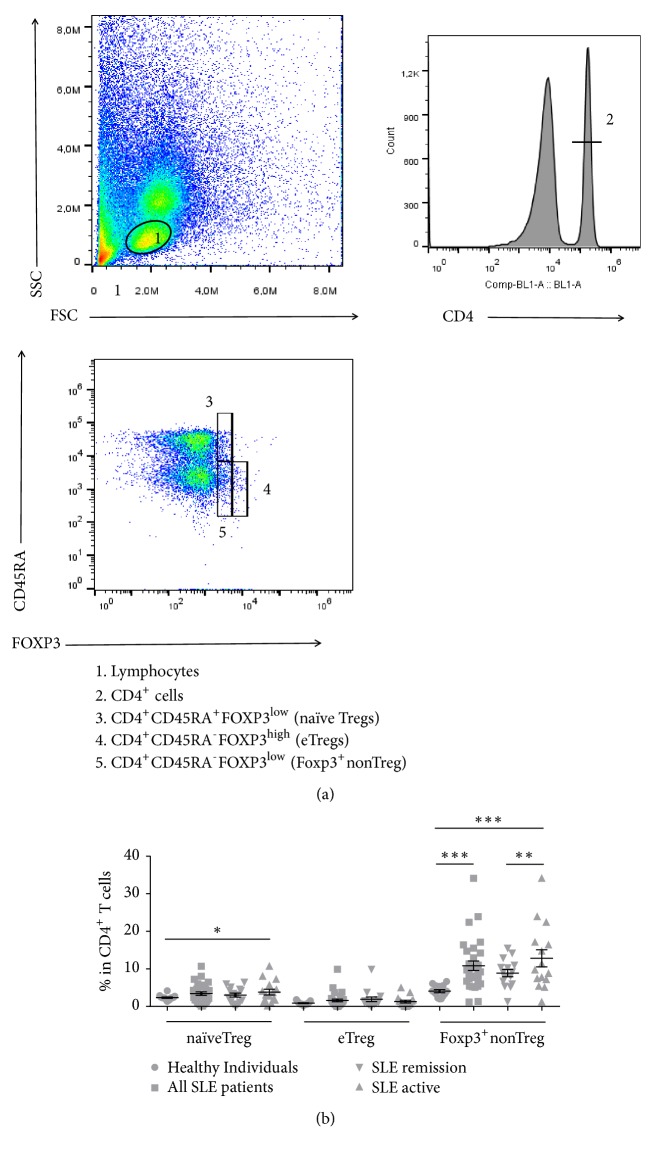
FOXP3^+^ Tregs subset phenotyping in patients with systemic lupus erythematosus (SLE) and healthy donors. (a) Gating strategy for Treg cells characterization in PBMCs: lymphocytes gate (1); TCD4^+^ lymphocytes (2); CD4^+^CD45RA^+^FOXP3^low^ (naïve Tregs) (3); CD4^+^CD45RA^−^FOXP3^high^ (eTregs) (4); CD4^+^CD45RA^−^FOXP3^low^ (Foxp3^+^nonTreg) (5). (b) naïveTreg, eTreg, and Foxp3^+^nontreg in TCD4^+^ cells. ^*∗*^p < 0.05; ^*∗∗*^p≤0.002; ^*∗∗∗*^p<0.0001.

Since SLE patients showed an increase in CD4^+^FOXP3^+^ Treg cells, we decided to investigate its subtypes. Based on the differential expression of the CD45RA marker and FOXP3 by CD4^+^ T lymphocytes, naïve Treg (Figure 2(a), gate 3), eTreg (Figure 2(a), gate 4), and nonTreg (Figure 2(a), gate 5) cells were evaluated. When analyzing all SLE patients, 3.6% (0.07-10.70%) of CD4^+^ T cells were naïve Treg and 1.14% (0.0-9.91%) eTreg. These values were higher than those found in healthy volunteers for the same subtypes (naïve Treg: 2.3%; 1.35-4.17% and eTreg: 0.64%; 0.25-1.8%), but only naïve Treg increase in SLE patients was significant (p=0.034). The most significant divergence was SLE Foxp3^+^nonTreg cells rates (9.18%, 1.19-34.10%) in contrast to that observed in healthy volunteers for this subtype (4.04%; 2,02-11.90%) (p<0.0001). In relation to SLE disease activity, naïve Treg or eTreg pattern did not vary between active and remission patients groups. However, patients with active disease showed the highest levels of Foxp3^+^nonTreg cells (9.7%, 1.19- 34.10%) compared to the group of patients in remission (9.19%, 1.32-15.50%) (p=0.002) (Figure 2(b)).

### 3.3. eTreg and Foxp3^+^nonTreg Subsets Indicate SLE Activity

To deepen the analysis, the influence of Treg cell subtypes on SLE activity, measured by SLEDAI through multiple linear regression analysis, was also evaluated. The eTreg, Foxp3^+^nonTreg, and naïveTreg together exerted an influence of 28.90% on SLEDAI score variability, with a high significance recorded by F-test (p=0.006). In this specific type of analysis, it was possible to detect that the eTreg subtype exerts an inverse influence on the severity of the disease (p=0.010), whereas Foxp3^+^nonTreg subtypes (p=0.003) is associated with an increased SLEDAI score. The correlation with naïve Treg and the disease activity was not significant (p=0.352). Also, the FOXP3^+^CD4^+^ phenotype (p=0.56) and FOXP3^+^CD4^+^ CD25^+^ CD127^−^ (p=0.52) had low influence on disease activity (1.47%), confirming our previous remarks. Additionally, this poor correlation was indicated by F-test (p=0.771), that showed no significance between these phenotypes and the disease activity evaluated by SLEDAI score (Table 2).

### 3.4. Proteinuria, Anti-dsDNA, and Rash Are Related to eTreg Reduction

Since the eTreg and Foxp3^+^nonTreg subtypes had the greatest influence on the SLE activity, we investigated specific SLEDAI clinical parameters that exerted influence on our sample correlated to these Treg subtypes (Table 3). We observed that an increase of proteinuria (p=0.006), anti-dsDNA (0.045), and rash (p=0.011) correlated inversely to the eTreg subtype frequency. Moreover, together, proteinuria, hematuria, pyuria, anti-dsDNA, complement, and rash may explain 32.55% (R squared) of population variability of the eTreg subtype on SLE patients (p=0.01) (Table 3). However, a similar analysis for the Foxp3^+^nonTreg subtype showed that the same set of clinical parameters did not significantly explain the variability of this subtype in our SLE sample (p=0.114) (data not shown). Therefore, Foxp3^+^nonTreg cells are the most significant SLE activity indicator identified, but only eTreg phenotype correlates with the specific disease clinical manifestations that we evaluated.

## 4. Discussion

SLE often evolves with hematological disorders including anemia, leukopenia, lymphopenia, and thrombocytopenia [28–30]. In our study, this was confirmed by the reduced proportion of CD4^+^T cells in SLE patients, even greater in the group with active disease. On the other hand, the percentage of FOXP3^+^CD4^+^ Treg cells was higher in patients, regardless of disease activity. Pan et al. (2012) [20] also showed higher CD4^+^FOXP3^+^ Treg cells in patients with SLEDAI> 5, while others studies did not show quantitative differences for the same phenotype [13, 19, 31].

Aiming to understand the role of Treg cells in SLE activity, other phenotypes of these cells have been explored in the disease. However, emerging analysis of CD4^+^CD25^+^CD127^−^ and CD4^+^CD25^+^FOXP3^+^ cells suggest that the latter is a promising SLE activity indicator, especially in renal involvement, and may facilitate the detection of Treg subsets with clinical relevance [21]. Therefore, we also investigated CD4^+^CD25^+^FOXP3^+^ phenotype, but we did not observe any variation according to disease activity (data not shown). For a more precise analysis of this phenotype, we also assessed CD127 expression, since according to previous reports the high CD25 expression and low CD127 expression analysis was equivalent to FOXP3 expression [32, 33]. However, CD4^+^CD25^+^FOXP3^+^CD127^−^ phenotype remained in constant proportions among patients in our sample, regardless of disease activity.

Although higher in SLE patients, CD4^+^FOXP3^+^ cells were not a good indicator of SLE disease activity. Therefore, we investigated its activation state according to differential expression of CD45RA and FOXP3, featuring the subtypes CD4^+^FOXP3^low^CD45RA^+^ (naïveTreg), CD4^+^FOXP3^high^CD45RA^−^ (eTreg), and CD4^+^FOXP3^low^CD45RA^−^ (Foxp3^+^nonTreg). Likewise Miyara [12] and Pan [20] groups, we also identified larger proportions of naïveTreg and Foxp3^+^nonTreg subtypes in SLE patients. Accordingly, we also detected a significantly higher percentage of Foxp3^+^nonTreg cells as a hallmark of SLE patients with an active disease. It is possible that the activation of this phenotype is a universal marker of the disease activity.

The Miyara group [12] was also able to observe a significant reduction in eTreg subtype among patients with active disease. Regardless of the convergence of data in different studies, it must be considered that they were conducted with patients in different degrees of disease activity. The nonuniformity in patient activity groups is a general limitation of studies with this disease.

Furthermore, these differences could depend not only on unclear definition of the Treg phenotype, as previously reported [21], since assessments of the same phenotypes provided contradictory results [34, 35]. These divergences probably arise from the different clinical parameters that constitute SLEDAI score for each sample of SLE patients investigated. This can justify, for example, why Pan and coworkers [20] were able to observe an increase in the frequency of naïve Treg cells related to the development of anti-dsDNA antibody in active SLE and we did not. This is consistent with the low frequency (36.66%) of anti-dsDNA antibody formation in our sample.

The analysis of naïve Treg, eTreg, and Foxp3^+^nonTreg together explains 28% of SLEDAI score variability in our sample. Evaluating the correlation coefficients for each subtype and their respective significance, we concluded that the eTreg subtype is inversely correlated with disease activity (p=0.010) while Foxp3^+^nonTreg (p=0.003) exerted a direct influence. Although naïveTreg frequencies exerts a direct influence, it was without significance for our sample (p=0.352). Additionally, we confirmed the low influence exerted by CD4^+^FOXP3^+^ and CD4^+^CD25^+^FOXP3^+^CD127^−^ cells frequencies on the SLEDAI score, both related only to 1.47% of the disease activity. It was also found that proteinuria (p = 0.006), anti-dsDNA antibody (0.045), and rash (p=0.011) were associated with eTreg cell reduction. Analysis of proteinuria, hematuria, pyuria, anti-DNA, complement, and rash may explain 32.55% eTreg frequency variations. Equivalent analysis for Foxp3^+^nonTreg did not identify specific clinical parameters related to frequency of this subtype, probably because the Foxp3^+^nonTreg phenotype can be correlated to multiple variables that go beyond those described in SLEDAI score.

Unlike naïveTreg and eTreg, Foxp3^+^nonTreg phenotype includes cells with a Th17 potential, that have no suppressive capacity, enhanced cell proliferation response, and exhibits strong IFN-*γ*, IL-17 and IL-2 production [12]. Therefore, this phenotype is consistent with disease progression and is in accordance with the predominance of a Th17 profile. In additional Th17 and Treg serum cytokyne analysis, we observed greater levels of IL-23 in SLE patients than in healthy subjects (Figure S 2.A). In supplementary analyses, we did not identify any correlation between serum levels of the investigated cytokines and Treg subsets or clinical characteristics (Figures S 2.B, C, D, and E).

Immunotherapies that target Treg cells and/or recovery of Treg cell homeostasis stand out in the search for more specific treatments for autoimmune diseases [5, 11, 24]. However, further studies are needed to optimize the characterization of these Treg cell subtypes, their functions, clinical correlations, and manipulations for self-tolerance reestablishment.

All SLE patients in this study were in treatment as shown in Table 1. This limitation of our study should be considered since such drugs may affect our results. Other studies in SLE context also shared this difficulty [10, 36]. However, we found that Treg subsets did not change in relation to the treatment adopted (p>0.05) (Figure S 3).

## 5. Conclusions

Our data demonstrated that eTreg and Foxp3^+^nonTreg frequencies correlate significantly with disease activity in systemic lupus erythematosus patients. The use of CD45RA as activation marker in CD4^+^FOXP3^+^ Treg cells allowed a more accurate analysis of a potential biomarker for active SLE, unlike conventional analysis based on CD25 and CD127 expression and in FOXP3^+^ CD4^+^ Treg cells. Although heterogeneity of Brazilian population is considerable [37, 38], we demonstrated that Foxp3^+^nonTreg subset is the most consistent indicator of SLE activity, which was confirmed by multiple linear regression analyses. In addition, we highlight Foxp3^+^nonTreg cells as an important tool for assessing disease activity in the search of new therapeutic strategies to reduce this phenotype and promote SLE remission.

## Figures and Tables

**Table 1:** Clinical and demographic parameters of SLE patients.

Number of patients	N = 30
Age (yrs), Mean (range)	38.17 ± 10.43 (19-61)
Sex, N (%)	
Female	28 (93.33)
Male	2 (6.66)
Disease duration (Months)	
Mean (range)	96 ± 72.94 (2 - 300)
Anti-dsDNA, N (%)	
Positive	11 (36.66)
Negative	19 (63.66)
Complement, N (%)	
Decreased	19 (63.66)
Normal	11 (36.66)
Treatment, N (%)	
Steroids	26 (86.66)
Antimalarial agents	26 (86.66)
Azathioprine	21 (70)
Mycophenolate mofetil	10 (33.33)
Disease activity (SLEDAI), N (%)	
Range	0-20
< 6	15 (50)
≥ 6	15 (50)
Nephritis, N (%)	
Active	10(33.33)
Inactive	20(66.66)

**Table 2:** Influence of Treg cells subtypes on SLEDAI score for sample.

SLEDAI	Coef.	Std. Err.	P > |t|	R-squared	Prob > F
eTreg	−1.206469	.435602	0.010	0.2890	0.0065
Foxp3^+^nonTreg	.3915982	.1208674	0.003		
naïveTreg	.3039881	.3204841	0.352		

^*∗*^Treg	−.3861103	.6703293	0.569	0.0147	0.7717
^*∗∗*^CD25	.998021	1.5654	0.529		

^*∗*^Treg: CD4^+^FOXP3^+^ cells/^*∗∗*^ CD25: CD4^+^FOXP3^+^CD25^+^CD127^−^ cells.

**Table 3:** Clinical parameters influence in eTreg cells frequencies of SLE patients.

eTreg	Coef.	Std. Err.	P > |t|	R-squared	Prob > F
Proteinuria	−1.271477	.4145096	0.006	0.3255	0.0163
Hematuria	.3083865	.7977254	0.703		
Pyuria	−.5732928	1.006636	0.575		
Anti-dsDNA	−2.084068	.9786697	0.045		
serum complement	1.581082	1.260822	0.223		
Rash	−1.476643	.5323137	0.011		

## Data Availability

The data, in free formats, used to support the findings of this study are available from the corresponding author upon request.

## Supplementary Materials

Supplementary Materials Figure S1: additional representative analysis of the positive region delimitation. eTreg (1), Foxp3^+^nonTreg (2), naïveTreg (3), and CD4^+^FOXP3^−^CD45RA^+^ T cells (4) (A). Differential PE-conjugated anti-FOXP3 antibody fluorescence intensity in eTreg, Foxp3^+^nonTreg, naiveTreg, and CD4^+^FOXP3^−^CD45RA^+^ T cells (4) subtypes (B). FMO control for FOXP3 delimitation (all fluorochromes minus FOXP3 marker) (C). Figure S2: Th17 and Treg related cytokines in the serum of SLE patients and healthy donors (A). Foxp3^+^nonTreg cells related to serum concentrations of IL-17 (B), IL-23 (C), TGF-*β* (D), and IL-10 (E) in SLE patients. All cytokines in SLE patients serum and healthy donors were measured using specific ELISA kits following the manufacturer's recommendations (eBiosciences or BD Biosciences). ^*∗*^p < 0.05. Figure S3: Treg subsets did not change in relation to the treatment adopted. (%) of the CD4^+^FOXP3^+^ Treg cells (A), CD4^+^FOXP3^+^CD25^+^CD127^−^ Treg cells (B), naïveTreg cells (C), eTreg cells (D), and Foxp3^+^nonTreg cells (E) in relation to the treatment adopted for SLE patients.
