# Supplementary material for: CD4+CD45RA−FOXP3low Regulatory T Cells as Potential Biomarkers of Disease Activity in Systemic Lupus Erythematosus Brazilian Patients
Source: Biomed Res Int. 2018 Jun 12;2018:3419565. doi: 10.1155/2018/3419565 (PMC6020667; doi:10.1155/2018/3419565)
Supplement: Supplementary Materials — Figure S1: additional representative analysis of the positive region delimitation. eTreg (1), Foxp3+nonTreg (2), naïveTreg (3), and CD4+FOXP3−CD45RA+ T cells (4) (A). Differential PE-conjugated anti-FOXP3 antibody fluorescence intensity in eTreg, Foxp3+nonTreg, naiveTreg, and CD4+FOXP3−CD45RA+ T cells (4) subtypes (B). FMO control for FOXP3 delimitation (all fluorochromes minus FOXP3 marker) (C). Figure S2: Th17 and Treg related cytokines in the serum of SLE patients and healthy donors (A). Foxp3+nonTreg cells related to serum concentrations of IL-17 (B), IL-23 (C), TGF-β (D), and IL-10 (E) in SLE patients. All cytokines in SLE patients serum and healthy donors were measured using specific ELISA kits following the manufacturer's recommendations (eBiosciences or BD Biosciences). ∗p < 0.05. Figure S3: Treg subsets did not change in relation to the treatment adopted. (%) of the CD4+FOXP3+ Treg cells (A), CD4+FOXP3+CD25+CD127− Treg cells (B), naïveTreg cells (C), eTreg cells (D), and Foxp3+nonTreg cells (E) in relation to the treatment adopted for SLE patients. [file 3419565.f1.pdf]

SUPPLEMENTARY MATERIAL

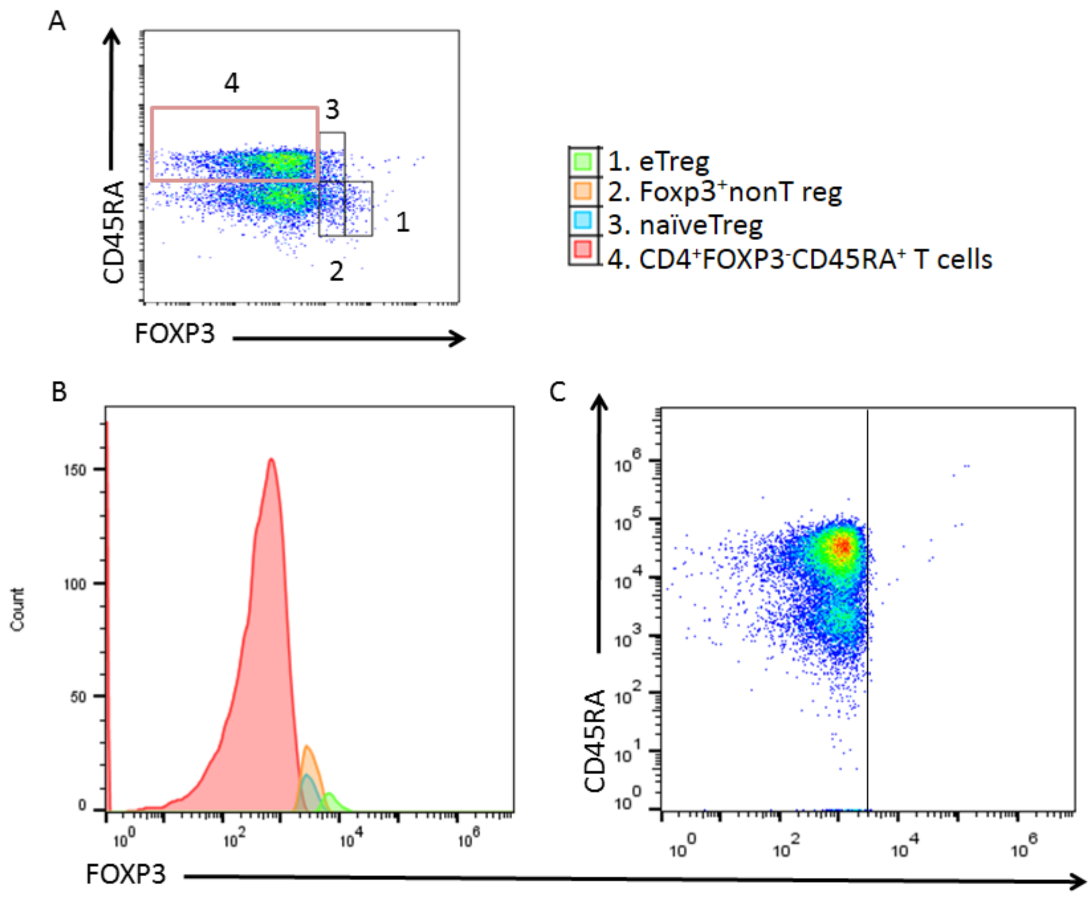

Figure S1: Additional representative analysis of the positive region delimitation. eTreg (1), Foxp3^{+}nonTreg (2), naïveTreg (3) and CD4^{+}FOXP3^{-}CD45RA^{+} T cells (4) (A). Differential PE-conjugated anti-FOXP3 antibody fluorescence intensity in eTreg, Foxp3^{+}nonTreg, naiveTreg and CD4^{+}FOXP3^{-}CD45RA^{+} T cells (4) subtypes (B). FMO control for FOXP3 delimitation (all fluorochromes minus FOXP3 marker) (C).

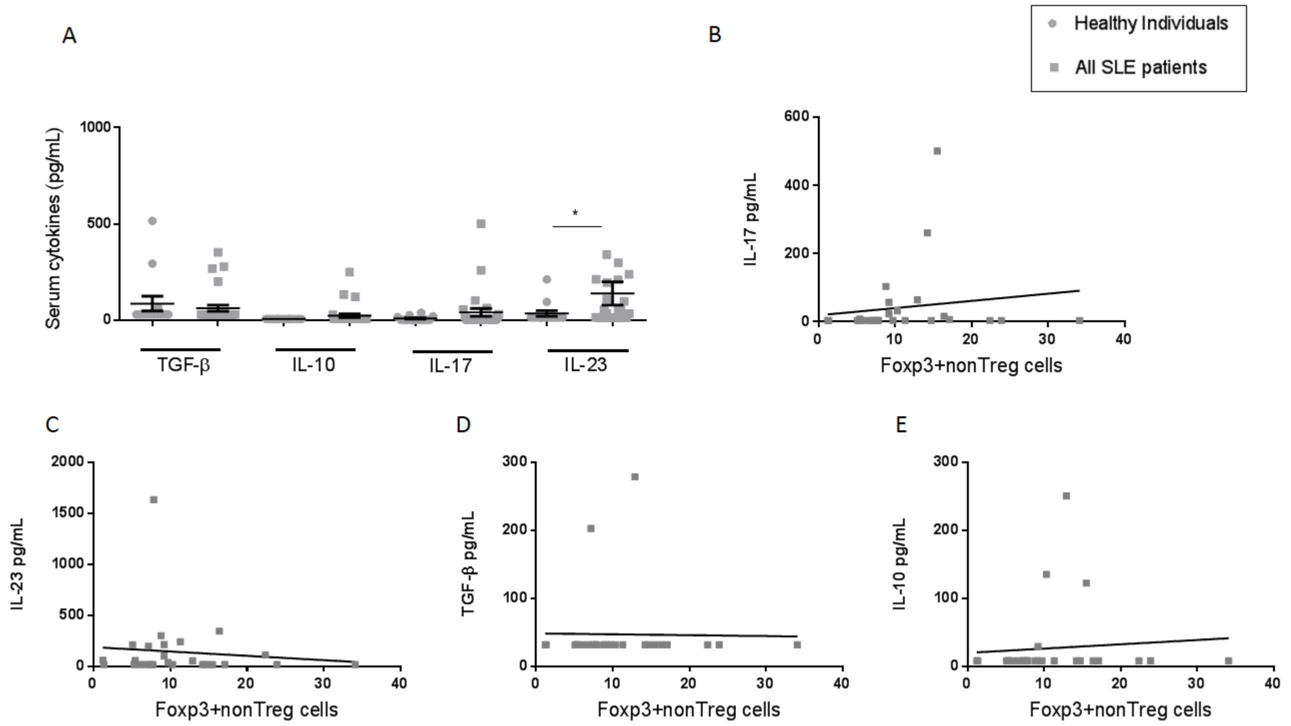

Figure S2: Th17 and Treg related cytokines in the serum of SLE patients and healthy donors (A). Foxp3^{+}nonTreg cells related to serum concentrations of IL-17 (B), IL-23 (C), TGF-β (D), IL-10 (E) in SLE patients. All cytokines in SLE patients serum and healthy donors were measured using specific ELISA kits following the manufacturer's recommendations (eBiosciences or BD Biosciences). *p < 0.05.

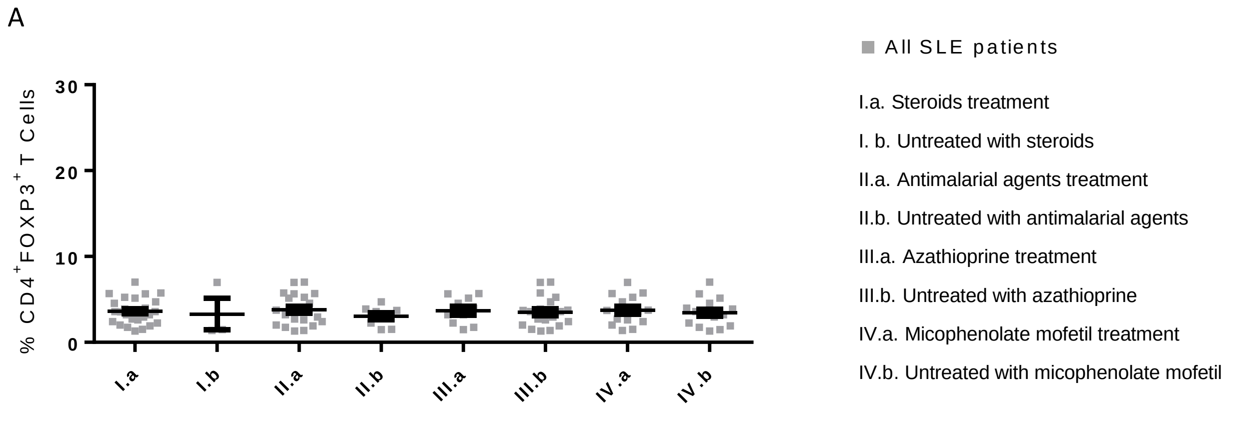

B

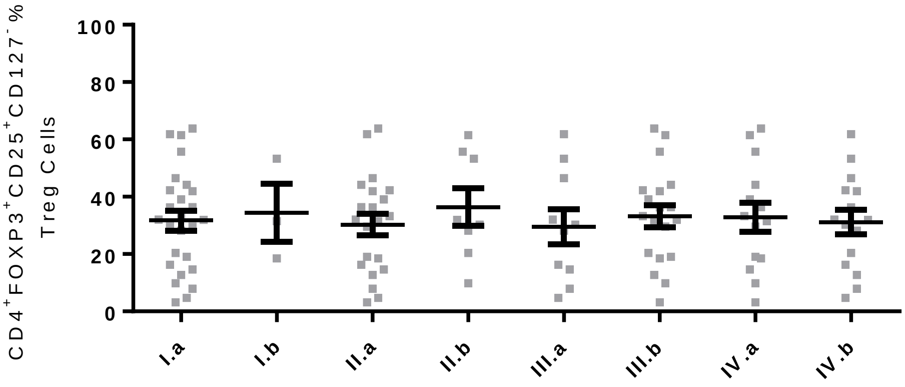
$CD4^{+}FOXP3^{+}CD25^{+}CD127^{-}$ %
Treg Cells
100
80
60
40
20
0
I.a
I.b
II.a
II.b
III.a
III.b
IV.a
IV.b

C

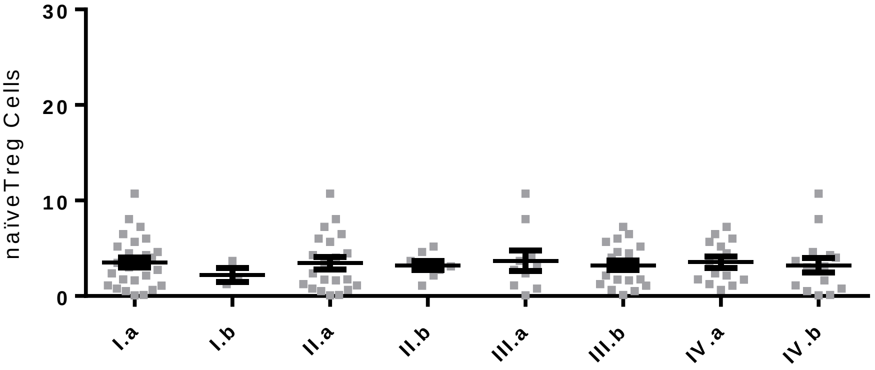
naïveTreg Cells
30
20
10
0
I.a
I.b
II.a
II.b
III.a
III.b
IV.a
IV.b

D

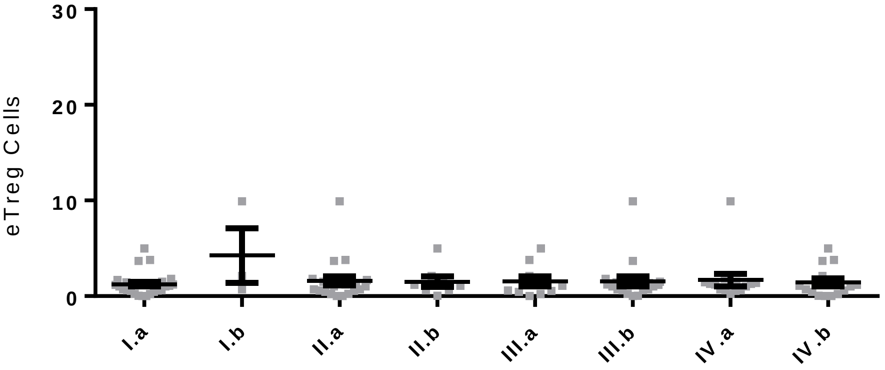
eTreg Cells
30
20
10
0
I.a
I.b
II.a
II.b
III.a
III.b
IV.a
IV.b

E

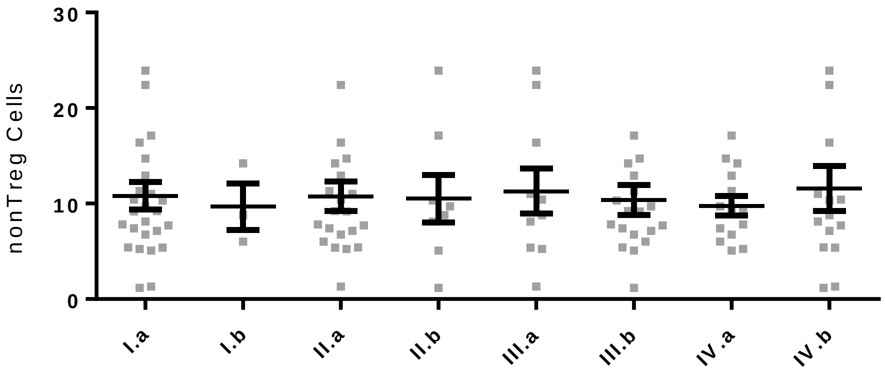

Figure S3: Treg subsets did not change in relation to the treatment adopted. (%) of the CD4$^+$FOXP3$^+$ Treg cells (A), CD4$^+$FOXP3$^+$CD25$^+$CD127$^-$ Treg cells (B), naïveTreg cells (C), eTreg cells (D) and Foxp3$^+$nonTreg cells in relation to the treatment adopted for SLE patients (E).
